# Measuring Progress towards a Circular Economy: A Monitoring Framework for Economy‐wide Material Loop Closing in the EU28

**DOI:** 10.1111/jiec.12809

**Published:** 2018-09-25

**Authors:** Andreas Mayer, Willi Haas, Dominik Wiedenhofer, Fridolin Krausmann, Philip Nuss, Gian Andrea Blengini

**Affiliations:** 1 Institute of Social Ecology (SEC), Department of Economics and Social Sciences, University of Natural Resources and Life Sciences (BOKU), Vienna, Austria; 2 German Environment Agency (UBA), Dessau‐Roßlau, Germany; 3 European Commission Directorate‐General Joint Research Centre Sustainable Resources Directorate, Unit D3 – Land Resources, Via Enrico Fermi 2749, 21027 Ispra, VA Italy

**Keywords:** circular economy, environmental pressures, industrial ecology, recycling, secondary materials, waste management

## Abstract

The concept of a circular economy (CE) is gaining increasing attention from policy makers, industry, and academia. There is a rapidly evolving debate on definitions, limitations, the contribution to a wider sustainability agenda, and a need for indicators to assess the effectiveness of circular economy measures at larger scales. Herein, we present a framework for a comprehensive and economy‐wide biophysical assessment of a CE, utilizing and systematically linking official statistics on resource extraction and use and waste flows in a mass‐balanced approach. This framework builds on the widely applied framework of economy‐wide material flow accounting and expands it by integrating waste flows, recycling, and downcycled materials. We propose a comprehensive set of indicators that measure the scale and circularity of total material and waste flows and their socioeconomic and ecological loop closing. We applied this framework in the context of monitoring efforts for a CE in the European Union (EU28) for the year 2014. We found that 7.4 gigatons (Gt) of materials were processed in the EU and only 0.71 Gt of them were secondary materials. The derived input socioeconomic cycling rate of materials was therefore 9.6%. Further, of the 4.8 Gt of interim output flows, 14.8% were recycled or downcycled. Based on these findings and our first efforts in assessing sensitivity of the framework, a number of improvements are deemed necessary: improved reporting of wastes, explicit modeling of societal in‐use stocks, introduction of criteria for ecological cycling, and disaggregated mass‐based indicators to evaluate environmental impacts of different materials and circularity initiatives. This article met the requirements for a gold – gold JIE data openness badge described at http://jie.click/badges.

http://jie.click/badges

**Supplementary Information:**

The online version of this article (doi:10.1111/jiec.12809) contains supplementary material, which is available to authorized users.

## Data Openness

bit.ly/JIE_data_trans

For information about the data openness badges, see bit.ly/JIE_data_trans

## Introduction

The idea of a more circular economy involves that the value and utility of products are extended and that production and consumption wastes are utilized as secondary resources, promising solutions and co‐benefits to a range of economic and environmental issues (Kirchherr et al. 2017; Winans et al. 2017). Thus, the concept of a circular economy (CE) has gained increasing attention from policy makers, industry, and academia (Geisendorf and Pietrulla 2017; Bocken et al. 2017a). Nevertheless, a broadly accepted and precise definition of a CE is still lacking; rather, the concept is applied in different ways by stakeholders, depending on their specific interests (Murray et al. 2017; Bocken et al. 2017b; Lieder and Rashid 2016; MacArthur 2013). For European countries, for example, Bocken and colleagues (2017a) have identified a focus of CE applications on business opportunities along with resource efficiency implications, while in China CE was developed around issues of pollution and in the context of China’s rapid growth. Also, positive employment effects are frequently mentioned as an important co‐benefit of the CE (Stahel 2016; Wijkman and Skanberg 2015; MacArthur 2013). Additionally, the environmental implications, benefits, and trade‐offs of a more circular economy are also widely debated (Pauliuk 2018; Geissdoerfer et al. 2017; Geisendorf and Pietrulla 2017).

The European Union embraced the concept of a CE as its key strategy towards a more sustainable use of natural resources. In 2015 the European Commission adopted a strengthened CE package that aims at maintaining the value of products, materials, and resources in the economy for as long as possible, and at minimizing the generation of waste as an essential contribution to the European Union’s (EU28) efforts to develop a sustainable, low‐carbon, resource‐efficient, and competitive economy (COM 2017). There, the transition from a “take‐make‐consume and dispose” economy towards a “recycle‐and‐reuse” economy was put into the center. In 2018, and as part of continuous efforts to transform the EU28 economy towards more sustainability, the European Commission adopted a range of CE‐related policy measures on, for example, plastics and improved legislation on waste or critical raw materials (European Commission 2018). Additionally, EU28‐wide monitoring efforts have been established.

While the basic idea of the CE is intuitive and convincing and the notion is widely used in policy documents, the assessment of progress towards a CE is an issue of ongoing debate (EASAC 2016; Haupt et al. 2017; Kovanda 2014; Hashimoto et al. 2009; Yuan et al. 2006). The majority of CE research has focused on individual products or specific substances within regions or nations, or at the global scale (Huysman et al. 2017). For some substances, mainly metals, the knowledge base on anthropogenic cycles has indeed improved in recent years (BIO by Deloitte 2015; Cullen and Allwood 2013; Graedel et al. 2013; Reck et al. 2008; Wang et al. 2007). A growing body of research has also focused on the company or industry level (Pauliuk 2018; BSI 2017; Lieder and Rashid 2016). However, comprehensive macro‐scale assessments of national‐level circularity, including derived policy indicators, are very rare (Nuss et al. 2017; Haas et al. 2015; Hashimoto et al. 2004).

Typically, two types of loop closing are distinguished in CE strategies (Haas et al. 2015; MacArthur 2013; UNEP 2012; Braungart et al. 2007; McDonough and Braungart 2003): socioeconomic loop closing by recycling waste materials as secondary material inputs and ecological loop closing by using renewable biomass. If these strategies lead to a reduction in the demand for primary resources, a more circular economy can lower pressures related to resource extraction, and by reducing the amount of wastes and emissions returned to the environment, it can mitigate pressures on the output side of societies’ metabolism (Geissdoerfer et al. 2017). To achieve a more sustainable economy, it is insufficient to only increase recycling and focus on (partial) improvements in the degree of circularity (Cullen 2017; Zink and Geyer 2017), but it is essential to also achieve absolute reductions in resource extraction and consumption, that is, to downsize the socioeconomic metabolism (Akenji et al. 2016). This has implications for the assessment of CE strategies and monitoring tools needed to measure both the degree of loop closing and the overall in‐ and outflows of societies’ metabolism (Krausmann et al. 2017a).

Thus, appropriate monitoring tools need to be able to capture different critical issues related to CE strategies. First, not all recycling activities are necessarily reducing overall resource demand, but they can result in problem shifting. Under certain circumstances, recycling indirectly may require more material and/or energy than the direct use of primary materials (Cullen 2017; Geyer et al. 2016; Behera et al. 2014). Second, a CE is often promoted as an environmentally friendly strategy to facilitate business opportunities and green economic growth, that is, a decoupling of resource use and environmental impacts from economic growth (Ekins 2002; Geng et al. 2013). Consequently, it needs to be critically assessed whether these promises can be realized (Cullen 2017; Zink and Geyer 2017). Third, in‐use stocks of manufactured capital are growing in most countries and require an increasing share of overall resource use, which substantially limits possibilities for loop closing (Krausmann et al. 2017b). Only if in‐use stocks are steady does a substantial closing of loops become possible because then end‐of‐life (EoL) flows from demolitions and discards can equal materials used for the maintenance and replacement of in‐use stocks (O’Neill 2015; Wiedenhofer et al. 2015). So far, only few studies have attempted to assess the CE at a more comprehensive level taking overall material flows into account (Nuss et al. 2017; Haas et al. 2015; Haupt et al. 2017; Kovanda 2014).

This article aims at contributing to the establishment of monitoring tools of material flows in a CE at the macro level, with the premise that a more circular economy should contribute to the reduction of environmental pressures instigated by resource use. Our proposal is to go beyond the level of individual products, substances, or industrial symbiosis but monitor progress towards a CE from an economy‐wide perspective at the national or higher scale. Only at this scale is it possible to also capture system‐wide effects such as displacement or rebound effects (Geyer et al. 2016) and to assess whether absolute reductions in resource use and waste flows were achieved. We aim at contributing to the debate of potentials and limitations of a more circular economy by developing a mass‐based monitoring framework for the European Union (European Commission 2018; Eurostat 2018). We based our framework upon previous attempts at system‐wide assessment of a CE at the global and European level by some of the authors (Haas et al. 2015). However, Haas and colleagues (2015) developed their assessment solely from economy‐wide material flow (ew‐MFA) data on material consumption and their uses. The circularity investigation by Nuss and colleagues (2017) focused on metals and nonmetallic minerals and nonenergy and nonfood biomass. Herein, we expand these efforts by, first, developing a comprehensive approach including all biomass, metals, nonmetallic minerals, and fossil energy carriers and, second, by reconciling officially available datasets published by the Statistical Office of European Communities (EUROSTAT) on material use, waste, and recycling (Eurostat 2017b, 2017c). Innovatively, we linked these datasets in a fully consistent and mass‐balanced way in order to achieve a systematic monitoring of resource use, waste, and recycling through the socioeconomic system. From this framework, we derived a set of indicators that measure the scale of input and output flows as well as socioeconomic and ecological loop closing.

The remainder of this article is organized as follows. The next section outlines the general accounting framework, methods and indicators for monitoring the CE, and a first sensitivity assessment. In the following sections, we then present the results in the form of Sankey diagrams of flows of materials through the EU28 economy in 2014 and discuss our findings. In the final section, we discuss the relevance and limitations of the proposed framework and sketch out the way ahead.

## Developing a Monitoring Framework for Material Loop Closing in the Circular Economy: Method, Data Sources, and Robustness

**Figure 1:**
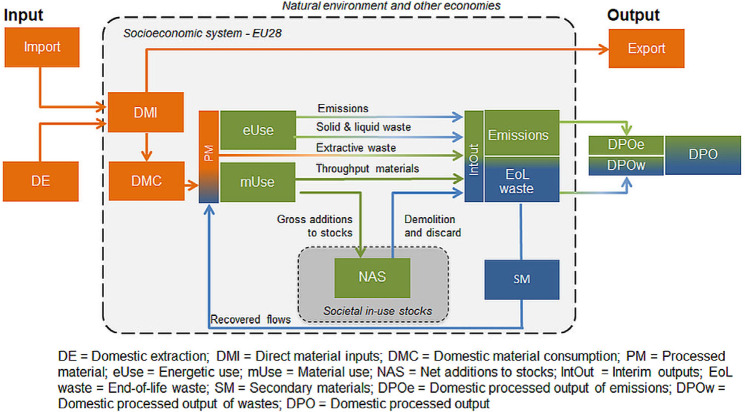
Framework and throughput indicators for an economy‐wide CE assessment. This framework applies from individual materials (e.g., DE of corn or iron) to aggregated material categories (e.g., PM of biomass, fossil energy carriers) to the total material level (e.g., total DE). Colors indicate data sources used: orange = official data from economy‐wide material flow accounts (Eurostat 2017c), blue = official waste and emissions statistics (Eurostat 2017b), green = mass‐balanced modeling. Please note that a shift from green to blue color indicates a combination of statistical data and modelling. See table S1 and figure S1 in the supporting information on the Journal’s website for an overview, definitions, and results for all flows and variables shown in figure 1. CE = circular economy.

We developed a CE monitoring framework at the macro level, built on previous research that proposed an expansion of ew‐MFA by including flows of secondary materials to allow for monitoring socioeconomic loop closing in national economies (Haas et al. 2015). Secondary materials refer to materials recovered through all forms of recycling, reuse, and remanufacturing but also downcycling (e.g., backfilling) or cascadic use. We built upon a systems and material perspective of the economy, and as a substantial advancement, we based the assessment as far as possible on statistical data from the official environmental reporting system of the EU (Eurostat 2017c, 2017b) and systematically mass‐balanced material inputs with waste flows reported in the different statistical sources. While recovered materials were reported in waste statistics and could be directly quantified, this was not possible for other CE strategies like extending product lifetimes, reusing and remanufacturing, or sharing. In our framework, these strategies would result in an increase of the service lifetime of in‐use stocks and potentially a stabilization of in‐use stock growth, as indicated by the net additions to stocks (NAS; figure 1). Thus, even though these strategies were difficult to measure directly, their effects on the size of inflows, additions to stock, and outflows can be substantial and are observable via this CE monitoring framework (figure 1).

### Quantifying and Tracing Material, Energy, and Waste Flows through the Socioeconomic System

The accounting framework shown in figure 1 traces materials by main material groups from their extraction to major uses within the socioeconomic system and towards discard and either material recovery or deposition to nature as wastes and emissions. In figure 1, the main physical stages of the flow of materials through the entire system are marked by throughput indicators, represented as boxes. These include the source of material inputs (e.g., domestic extraction, imports), major material transformation processing stages within the system (e.g., processed materials; energetic use; material use, in‐use stocks of materials; waste treatment; EoL waste) and the destination of outflows (e.g., exports, domestic processed output to the environment). Flows of materials are displayed as arrows between these boxes; the colors of flows indicate the type of data source (e.g., orange for ew‐MFA, blue for waste statistics).

Direct material input (DMI) into the socioeconomic system comprises materials that were extracted from the domestic environment (domestic extraction [DE]) and imports of raw materials and manufactured products. Exports to other economies were deducted to calculate domestic material consumption (DMC). Processed materials (PMs) were defined as the sum total of DMC and secondary material (SM) inputs.

PMs were allocated to either energetic or material use (see table S2 in supporting information S1 available on the Journal’s website for detailed allocation tables). Energetic use (eUse) not only comprises materials used to provide technical energy (fuel wood and biofuels) but also feed and food, the primary energy sources for livestock and humans (Haberl 2001; Krausmann et al. 2016). The majority of all fossil energy carriers were allocated to eUse with the exception of, for example, petrochemical feedstock. All other materials were assigned to material use (mUse). mUse comprises all metal ores and metals and nonmetallic minerals, as well as those fractions of fossil and biomass materials not used for energy provision. The biomass fractions assigned to material use include industrial roundwood, crops for uses other than food, and feed including seeds and most harvested crop residues.

mUse was split into extractive waste, materials used for stock building (i.e., gross additions to in‐use stocks of materials [GAS]), and throughput materials (Krausmann et al. 2017b; Haas et al. 2015; Lauk et al. 2012). Extractive waste refers to waste material that occurs during early stages of the processing of domestically extracted ores (European Parliament, Council of the European Union 2006) and directly goes from PM to interim output (IntOut). Stock building materials comprise all materials that accumulate in buildings, infrastructures, or durable goods with a lifetime of more than one year (e.g., concrete, asphalt, or steel). The share of stock‐building materials in mUse was estimated based on information from industry and production statistics, results from material flow studies and assumptions (see table S2 in supporting information S1 on the Web). Throughput materials comprise materials that do not accumulate in in‐use stocks, and can be split into two types of materials: first, materials used deliberately in a dissipative way such as salt or fertilizer minerals, and losses that occur during material processing (wastage, not reported in waste statistics); and second, short‐lived products such as packaging or newspaper, manufacturing wastes, and food waste (reported in waste statistics).

All materials that are neither added to stocks nor recycled are converted into gaseous, solid, or liquid outputs within the year of extraction. Together with demolition and discard from in‐use stocks that have reached the end of their service lifetime, these outflows were denoted as interim outputs (IntOut) in figure 1. IntOuts were split into emissions, comprising all gaseous emissions (e.g., carbon dioxide [CO_2_], sulfur dioxide [SO_2_], methane [CH_4_]) including water vapor and into EoL waste, including all solid (and liquid) outputs. Information on outflows was either sourced from Eurostat waste statistics or modeled and mass‐balanced with input flows (see below and supporting information S1 and supplementary data S2 on the Web for details). Emissions cannot be recycled and go straight into domestic processed output (DPO). A fraction of total EoL waste, reported as RCV_B – (recovery other than energy recovery—backfilling*)* and RCV_O (recovery other than energy recovery—except backfilling) in Eurostat (2017b) waste statistics, is reentering socioeconomic processes as secondary materials. Note that imported or exported secondary materials (e.g., scrap, waste paper) were not explicitly accounted for as secondary materials but included in trade flows; they are therefore also not reflected in circularity indicators. The remaining EoL waste (after subtracting SM) is returned to the environment as DPO waste and either landfilled, incinerated, or deliberately applied (e.g., manure, fertilizer). DPO emissions and DPO waste together are DPO.

### Closing the Mass Balance between Material Inputs and Outputs of Wastes and Emissions

To close the material balance between input and output flows we combined data from statistical reporting (i.e., from ew‐MFA and waste statistics) with modeling. This was done separately for eUse and for the mUse components in two balancing calculations. The following equations summarize the mass balancing for eUse (equation 1) and mUse (equation 2).

1 $$ DPO\kern0.44em emissions= eUse- solid\kern0.32em and\kern0.32em liquid\kern0.32em wastes $$

2 $$ {\displaystyle \begin{array}{ccc}\hfill Demolition\kern0.32em and\kern0.32em discard& =& EoL\kern0.32em waste\kern0.32em from\kern0.32em mUse\hfill \\ {}& & - throughput\kern0.32em materials\kern0.32em in\kern0.32em waste\hfill \end{array}} $$

#### eUse Balancing

We assumed that all materials used to provide energy were converted into DPO emissions (including water vapor) and solid waste within the year of extraction. We used data for solid waste from combustion reported in waste statistics and estimated the amount of solid waste from human and animal metabolism (excrements) by applying appropriate coefficients reflecting the nondigestible fraction of food and feed intake. DPO emissions were then calculated as the difference between eUse and the outflow of solid waste. Note that so‐called balancing items (oxygen uptake from air during combustion and water consumed by humans and livestock) were excluded. This means that all outflows from eUse include only the materials contained in actual inputs as comprised in PM (e.g., CO_2_ or SO_2_ in terms of C or S content; excrements at the average water content of food and feed intake). Closing the mass balance for eUse in this way implies that all inaccuracies in statistical data and assumptions that result in inconsistencies between input and output flows accrued in DPO emissions (DPOe). For the combustion of fossil energy carriers we cross‐checked the calculated DPO emissions with data from emission statistics. We found that our estimate is roughly 6% above emission statistics, which is a reasonably good fit, considering that emission statistics do not provide a comprehensive coverage of all emissions (see table S5 in supporting information S1 on the Web).

#### mUse Balancing

Due to a lack of knowledge of actual in‐use stocks, we used the following approach to close the material balance: In a first step, a consistent split of total EoL waste from mUse into waste flows resulting from discard and demolition and throughput materials was required. Total EoL waste from mUse was derived from waste statistics. While waste statistics report information on construction and demolition waste, this waste flow was not fully consistent with EoL waste from discard and demolition, which also contains waste flows from discarded long‐living products such as furniture, cars, or electric appliances. In a second step, we calculated the amount of discard and demolition as the difference between EoL waste from mUse reported in waste statistics and the fraction of throughput materials (i.e., materials with a life span < 1 year) in mUse (e.g., waste from packaging, paper, food waste, etc.). In a third step, NAS were calculated as the difference between additions to stocks and discard and demolition. Closing the mass balance in this way implies that all inaccuracies in statistical data and assumptions that result in inconsistencies between input and output flows for mUse accrue in demolition and discard flows as residual flow category, and consequently in the value for NAS. Since no information on NAS in the EU28 was available, a cross‐check with independent data was not possible. Krausmann et al. (2017b) have estimated NAS in industrial countries in 2014 to amount to 6.7 tons per capita per year (t/cap/yr). For the EU28 we arrived at 5.2 t/cap/yr. Given that the two estimates refer to different country groups and apply different methods, we conclude that our estimate is in a plausible range.

All flows and indicators were calculated for the four main material groups distinguished in ew‐MFA: nonmetallic minerals, metal ores and metals, fossil energy carriers, and biomass. The calculation at the level of material groups was challenging because waste statistics of Eurostat [*Regulation (EC) No 2150/2002;* see European Commission and Eurostat (2013)] follow a classification that refers to economic sectors and activity (NACE classification), different collection systems, and/or hazard potential. Waste materials reported in one category typically comprise multiple material categories in ew‐MFA, which required an allocation of output to input flows. Waste flows reported in waste statistics needed adjustments to the system boundaries used in ew‐MFA to ensure that input and output flows can be mass balanced (see table S4 in supporting information S1 on the Web for detailed allocation tables).

#### Deriving Mass‐Based Indicators for a Circular Economy

Indicators highly depend on the underlying definition of a CE. Here, we follow a definition that was first developed by Braungart and colleagues (2007) and summarized by the Global Energy Outlook (UNEP 2012) and defines a CE as an economy in which material flows are made up either of biological materials, which after discard are integrated into ecological cycles, or of materials designed to circulate within the socioeconomic system (MacArthur 2013). Based on this general definition, we developed a set of six indicator pairs, which allows us to measure progress towards a CE in terms of closing material cycles (table 1). The indicators presented here are based on ew‐MFA principles and build on proposals from previous research (Nuss et al. 2017; Haas et al. 2015; Hashimoto et al. 2004; Kovanda 2014). We distinguished between scale indicators, which provide measures for the overall size of the socioeconomic metabolism (O’Neill 2015), and circularity rates, which measure socioeconomic and ecological cycling relative to input and output flows. Providing independent measures for flows on both the input and output sides is necessary because of the delaying effect that in‐use stocks of materials have on output flows.

**Table 1:** Mass‐based circular economy indicators for the EU28 in 2014 where scale indicators measure the absolute size of input and outputs flows in tons and circularity rates measure socioeconomic and ecological cycling relative to input and output flows in percentage^1^

	Dimension	Input‐side indicator	Output‐side indicator
*Scale indicators (t)*	In‐ and output flows	*Domestic material consumption* *** DMC***	*Domestic processed outputs* ***DPO***
	*Consumption based perspective*	*Raw material consumption* ***RMC***	*n.a*.
	Interim flows	*Processed materials* ***PM*** = DMC + secondary materials	*Interim outputs* ***IntOut*** = EoL waste + DPO emissions
*Circularity rates (%)*	*Socioeconomic cycling SC*	*Input socioeconomic cycling rate* ***ISCr*** = Share of secondary materials in PM	*Output socioeconomic cycling rate* ***OSCr*** = Share of secondary materials in IntOut
	*Ecological cycling potential EC*	*Input ecological cycling rate* potential ***IECrp*** = Share of DMC of primary biomass in PM	*Output ecological cycling rate* potential ***OECrp*** = Share of DPO biomass in IntOut
	*Non‐circularity NC*	*Input non‐circularity rate* ***INCr*** = Share of eUse of fossil energy carriers in PM	*Output non‐circularity rate* ***ONCr*** = Share of eUse of fossil energy carriers in IntOut

*Note*: n.a. = not applicable.

We used three pairs of indicators to measure the scale of material and waste flows: DMC measures all materials directly used in a national production system and is regarded as proxy for the aggregated pressure the economy exerts on the environment (Krausmann et al. 2017a). DPO measures the total amount of outflow of wastes and emissions from a national economy. In order to be able to capture displacement effects related to imports and exports, we include a consumption‐based indicator in the framework. For this purpose, Eurostat (2017a) provides the indicator raw material consumption (RMC), which is similar to the material footprint (Wiedmann et al. 2015) and measures global material use associated with domestic final consumption (Krausmann et al. 2017a). The final pair of scale indicators takes the flow of secondary materials into account, which is not presented in conventional ew‐MFA indicators: On the input side, the indicator PM measures the sum total of DMC plus the input of secondary materials, and on the output side, IntOut measures wastes and emissions before materials for recycling and downcycling are diverted. Even in industrial countries, stocks are growing and interim outflows in a given year are much smaller than the amount of PM in that year, which further inhibits loop closing at present, producing a delaying effect for potential recycling of these materials after their lifetime has ended in the future.

As indicators for the degree of loop closing that has been achieved, we propose three pairs of circularity rates, which measure material flows relative to interim flows PM and IntOut. The socioeconomic cycling rates measure the contribution of secondary materials to PM (input socioeconomic cycling rate [ISCr]) and the share of IntOut that is diverted to be used as secondary materials (output socioeconomic cycling rate [OSCr]). For biomass, derived circularity indicators are more intricate. Due to the absence of a clear definition and recognized criteria for sustainably produced biomass, as well as a lack of related data, we use the share of primary biomass (i.e., biomass DMC) in PM for the input ecological cycling rate potential (IECrp) and the share of DPO from biomass in IntOut for the output ecological cycling rate potential (OECrp). Because ecological cycling is a crucial part of CE strategies, data and adequate indicators have to be developed so that socioeconomic and ecological cycling rates indicate the overall circularity of an economy. Finally, the noncircularity indicators measure the share of eUse of fossil energy carriers in PM and IntOut, thus quantifying the share of material flows that do not qualify neither for socioeconomic and ecological loop closing. Due to unreliable information on dissipation rates of fertilizers or salt for deicing roads, for example, we did not allocate these materials to noncircularity flows. The difference between 100% and the sum total of the three circularity rates serve as a measure for the unexploited potential for socioeconomic cycling.

The indicators proposed herein were developed to detect and monitor economy‐wide improvements and trade‐offs for economy‐wide circularity. Since specific materials or material categories are interconnected (Bleischwitz et al. 2018), enhancing circularity for one material has an impact upon the use of other materials and energy. In the case of plastic, construction minerals, or metals, recycling might mean high energy investments in recycling operations, infrastructure, and transport. Consequently, increasing recovery rates, for example, for plastic waste, might reduce overall circularity through higher energy use. Thus, we used the shares of socioeconomic, ecological, and noncircular flows in PM and IntOut as circularity indicators that are able to monitor system‐wide implications of circularity initiatives.

### Validation and Sensitivity: How Robust Are the Derived Circular Economy Indicators?

Generally, for ew‐MFA a systematic assessment of uncertainty is in its infancy (Patrício et al. 2015; Laner et al. 2014). It has been shown that material flow accounts using different sources and estimation procedures typically differ in their results for global DE by approximately 5% to 20% (Schandl et al. 2017; Krausmann et al. 2017a; Fischer‐Kowalski et al. 2011). For waste statistics, harmonization of data reporting across countries is a major challenge, and data quality differs across EU28 countries (Tisserant et al. 2017; Nicolli et al. 2012).

**Figure 2:**
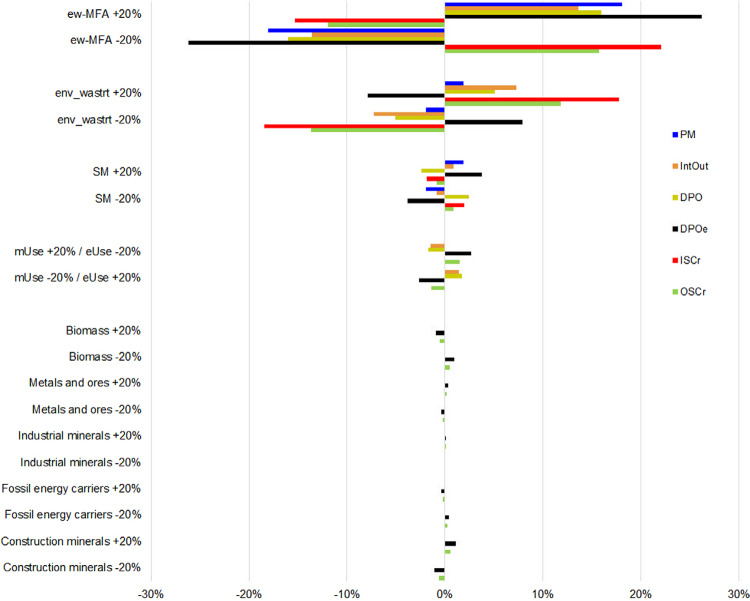
Results for the one‐at‐a‐time (OOAT) ± 20% sensitivity tests of the CE assessment for four scale and two circularity indicators. Due to the modeling framework architecture, we performed sensitivity e for industrial minerals and construction minerals, respectively. Results are presented as the relative change of each indicator due to the ±20% sensitivity test for the identified main sources of uncertainty. For detailed results, see table S3 in supporting information S1 on the Web. Ew‐MFA = EUROSTAT ew‐MFA dataset (Eurostat 2017c). env_wastrt = EUROSTAT waste treatment statistics (Eurostat 2017b), mUse = material use, SM = secondary materials.

We employed a one‐at‐a‐time sensitivity assessment (Saltelli et al. 2000) to quantify the significance of variations in all major data sources and allocation steps used in the CE assessment for four scale indicators (PM, IntOut, DPO, DPOe) and two circularity indicators (ISCr and OSCr) from our framework and applied a ±20% sensitivity test on the underlying statistical data (a–c) and allocation schemes (d–e). We conducted such tests for (a) the ew‐MFA data, (b) waste statistics, (c) secondary materials, (d) the allocation between energetic and material use, and (e) the matching of waste flows to ew‐MFA categories (figure 2).

We found that all indicators were most sensitive to changes in the underlying statistical data, with material flow data causing the largest sensitivities (sensitivities a–c, see figure 2). DPOe showed the highest and same directional sensitivity with ±26% (±20% variation in ew‐MFA data), followed by lower sensitivities for PM, DPO, and IntOut. A ±20% variation in waste statistics caused sensitivities less than 10% for all scale indicators, and ±20% variations in the amount of secondary materials showed lover sensitivities of less than 4%. ISCr and OSCr responded nonlinearly to changes in input data, again in declining magnitude from sensitivities a through d (except sensitivity d for OSCr).

All indicators were much less sensitive to changes in the allocation schemes (sensitivities d–e). The output‐related scale indicators responded with ±1.4% to ±2.6% to a ±20% change in the assumptions used for the allocation of PM to eUse and mUse, with the OSCr showing a sensitivity less than 1.5%. All other allocation sensitivities were below ±1.1% for the selected indicators.

Overall, the results of the one‐at‐a‐time sensitivity test indicated that the CE indicators and main results of the assessment are quite robust against variations in the allocation of waste flows to main material categories for the assessed material flows and indicators. The strongest relative changes in the assessed flows and indicators relate to the quality of input data.

## Results

### Material Flows through the EU28 Economy

**Figure 3:**
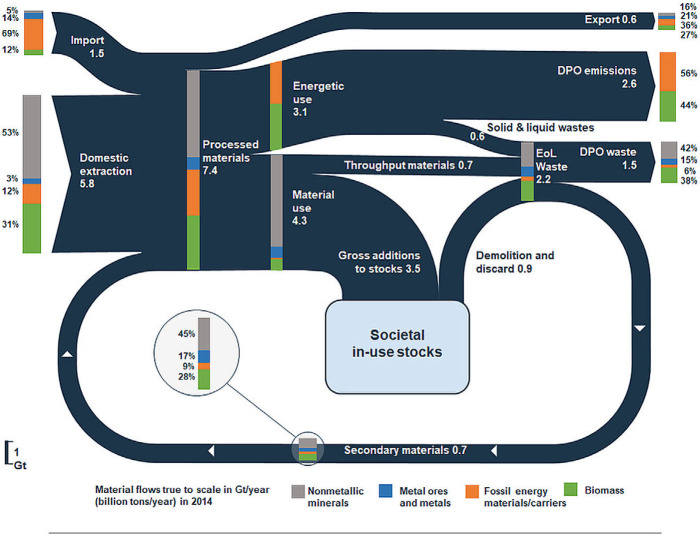
Material flows through the EU28 economy in 2014. In this Sankey diagram, the width of the arrows is proportional to the size of material flows (dark blue); the numbers show the size of the material flows in Gt/yr and the bars their composition (share of four main material groups in %). Note that numbers may not always sum up to total due to rounding. EU28 = European Union; Gt/yr = gigatons per year.

Applying the CE monitoring framework to the EU28 in 2014 shows that 5.8 Gt (1 gigaton = 10^9^ tons) of raw materials, which were used in the EU, originated from domestic extraction, 0.9 Gt from net imports, and 0.7 Gt from secondary materials (figure 3). Of the 7.4 Gt of PM, 3.1 Gt were used to provide energy and 4.3 Gt for material use. Throughput materials including extractive waste accounted for 0.7 Gt, while the majority of 3.5 Gt were used to expand and maintain material in‐use stocks of buildings, infrastructure, and other long‐lived material products. Overall, 2.6 Gt have been net additions to in‐use stocks. Thus, nearly one third of all processed materials in 2014 increased the material stocks in the EU28 and will remain in use for years to decades, thereby shaping future waste flows and potentials for closing material loops, but also requiring energy use for their operation. A total of 2.2 Gt of EoL wastes resulted from demolition and discard, throughput materials, and solid wastes from energetic use, of which one third were recovered and reused as secondary materials. The remaining two thirds were landfilled or incinerated and thus eventually released to the environment. DPO emissions to air accounted for 2.5 Gt, of which 1.1 Gt originated from food, feed, and biomass energy, and 1.4 Gt from fossil energy carriers.

**Figure 4:**
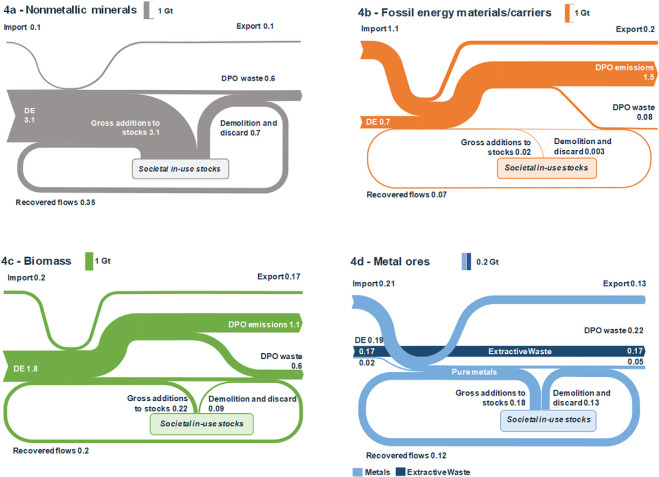
Material flows through the EU28 economy in 2014 for four main material groups in Gt/yr. (a) Nonmetallic minerals, including both construction and industrial minerals; (b) fossil energy carriers; (c) biomass; (d) metal ores and metals (note that the scale for metal ores is factor 5 lower). Balancing flows of oxygen and water are not included. Note that numbers may not always sum up to total due to rounding. DE = domestic extraction, DPO = domestic processed output; EU28 = European Union; Gt/yr = gigatons per year.

Figure 4a–d show results for the four main material categories (see supporting information S1 and supplementary data S2 on the Web for additional results). The largest material flow in the EU28 were nonmetallic minerals, which amounted to 3.5 Gt or 50% of all processed materials and are shown in figure 4a. The majority of nonmetallic minerals were used to maintain and expand societal in‐use stocks, resulting in 2.4 Gt of net additions to material stocks. Of the total EoL waste from nonmetallic minerals, one third (0.35 Gt) were recovered, equivalent to a material‐specific output cycling rate of 33.8%, and an input cycling rate of 9.5%, caused by the large net additions to stocks.

Only a small fraction (0.05 Gt) of processed fossil energy carriers were used for nonenergetic purposes (figure 4b). Of the 0.15 Gt of solid and liquid residues from fossil energy materials/carriers use, 42% were recovered, mainly bitumen and plastics, although the material‐specific output circularity is only 4.1%, due to the majority of fossil energy carriers used for energy provision. The 1.7 Gt of eUse of fossil energy carriers did not qualify for socioeconomic cycling and lead to 1.5 Gt of emissions to air (including water vapor but excluding oxygen from air), which are a major driver for anthropogenic climate change (IPCC 2013).

For biomass (figure 4c), we found that 90% of the 2 Gt PM were sourced from extraction within the EU28 in 2014, of which roughly one fifth were used for material purposes; most importantly, wood, with 0.3 Gt. 78% of all biomass (1.6 Gt), was used as energy source to feed livestock and humans and to provide technical energy, leading to 1.1 Gt of emissions. A total of 0.2 Gt (or 26% of EoL waste of biomass) were recovered, leaving 0.6 Gt DPO waste flows to be returned to the environment through landfills or incineration.

Metal ores and metals are materials of high economic and thermodynamic value. Additionally, they also have high strategic importance, given that 90% (0.21 Gt) of all processed metals were imported from other world regions (figure 4d). Domestic extraction of metals contributed only 0.02 Gt to the PM of 0.22 Gt but generated 0.17 Gt of extractive waste. The majority of processed metal ores and metals, of which nearly one third were secondary materials, have been integrated into material stocks.

### Circularity Indicators

**Figure 5:**
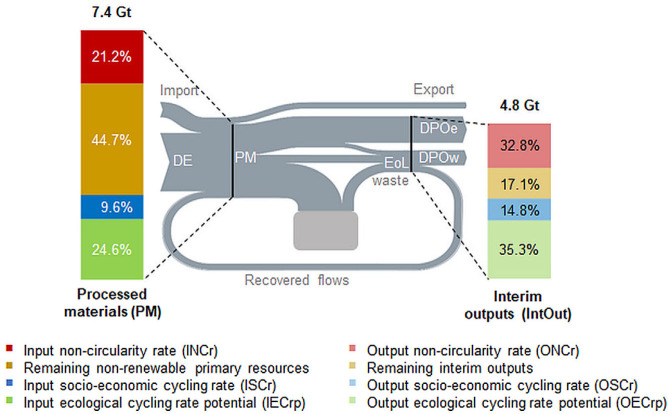
Input‐ and output‐side CE indicators. Left bar depicts processed materials; right bar depicts interim outputs. Percentage denotes the share in relation to processed materials (left bar) and interim outputs (right bar).

In 2014, 65% (4.8 Gt) of PM were converted into IntOut. The remaining 35% were added to in‐use stocks of buildings, infrastructure, and durable goods, which have grown in 2014 by 2.6 Gt (NAS) (figure 5 and table 2). Even though nearly one third of the total EoL waste was recovered and used as secondary resources, the OSCr remained low, at only 14.8%. The input socioeconomic cycling rate, measuring the recycled and downcycled materials that were reprocessed as secondary material inputs into the domestic economy was even lower, at 9.6%. Ecological cycling, although indicated only as theoretical potential, was comparatively high; the IECrp, indicating the maximum share of PM that qualifies for ecological cycling, was 24.6%; the OECrp was even higher, at 35.3%. The high significance of fossil energy carriers in the EU28 primary energy supply, flows that cannot be recycled or reused, led to an input noncircularity rate (INCr) of 21.2%, and an output noncircularity rate (ONCr) of 32.8%.

**Table 2:** Mass‐based circular economy indicators for the EU28 in 2014

	Input‐side indicator		Output‐side indicator	
Scale indicators (Gt/yr)	Domestic material consumption (DMC)	6.7	Domestic processed outputs (DPO)	4.1
	Raw material consumption (RMC)	7.2	NA	
	Processed materials (PM)	7.4	Interim outputs (IntOut)	4.8
Circularity rates (%)	Input socioeconomic cycling rate (ISCr; share of PM)	9.6	Output socioeconomic cycling rate (OSCr; share of IntOut)	14.8
	Input ecological cycling rate potential (IECrp)	24.6	Output ecological cycling rate potential (OECrp)	35.3
	Input non‐circularity rate (INCr)	21.2	Output noncircularity rate (ONCr)	32.8

*Note*: Indicators differentiate between input‐ and output‐side and in the scale of flows and related circularity rates. Scale indicators presented in Gt/yr, circularity rates in percentage.

Gt/yr = gigatons per year; NA = not applicable.

While our assessment provided a snapshot for a single year, Eurostat material flow analysis and waste statistics allow deriving some rough indications on the direction of the development. For the years 2010 and 2012, consistent information on DMC and the flow of recycled and downcycled materials was available, which was used to calculate the ISCr (table 3). We found a slight decrease in DMC and a more or less stable flow of secondary materials, which resulted in a slight growth in ISCr between 2010 and 2014. Slight progress towards a CE was rather due to reductions in material input, while socioeconomic cycling showed little improvement. Providing indicators for ecological loop closing was more intricate, but the slightly increasing share of biomass in DMC indicates a growing ecological cycling potential.

**Table 3:** Comparison of DMC, secondary materials (SM), processed materials (PM), input socioeconomic cycling rate (ISCr), and share of biomass in DMC for 2010, 2012, and 2014

	2010	2012	2014
DMC^a^ (Gt)	7.0	6.8	6.7
Secondary materials^b^ (Gt)	0.72	0.73	0.71
PM (Gt)	7.7	7.5	7.4
ISCr = Secondary materials/PM (%)	9.3%	9.7%	9.6%
DMC biomass/DMC (%)	23.2%	24.4%	27.2%

^a^Data source for 2010 and 2012: Eurostat 2017c.

^b^Data source for 2010 and 2012: Eurostat 2017b.

Gt = gigatons.

## Discussion

### Assessment of Circularity

We investigated the status quo of the CE in the EU28 in 2014 and developed a set of headline indicators on the scale and circularity of material and waste flows. Of 7.4 Gt of PMs, 6.7 Gt were primary resources and led to 4.8 Gt of IntOuts. All circularity indicators showed higher rates when compared to outflows, and these differences were due to the growth of in‐use stocks in the EU28 economy. Nearly one tenth of all PMs (9.6%), and 14.8% of all IntOuts were secondary resources. Acknowledging the caveats in calculating and interpreting ecological cycling rates, we found potentials of 24.6% and 35.3% for input and output ecological cycling rate potentials, respectively. The low socioeconomic loops we found in our study are also lower than in Haas and colleagues (2015), who estimated an ISCr of 13% for the EU27 in 2005 (see table S6 in supporting information S1 on the Web for a more detailed comparison). This study did not use data from actual waste statistics but was based on a simple leaching model for outflows from in‐use stocks and assumed that all abandoned stocks after EoL turn into demolition and discard waste, thus ignoring the issue of hibernating stocks. This resulted in an overestimation of waste flows and, by applying recycling rates rather than statistical data, to an overestimation of circularity rates.

Overall, input and output socioeconomic cycling was surprisingly low, considering the fact that the EU28 has strict waste regulations, elaborate waste collection and recovery systems, and high material category‐specific recovery rates that range from 25% for biomass to 70% for metals. While it is important to further improve the recycling and downcycling of EoL waste, our results emphasize that achieving a CE goes far beyond increasing reuse and recycling. Major obstacles for substantially increasing socioeconomic cycling are the ongoing expansion of in‐use stocks and the high share of noncircular fossil energy carriers in PM (INCr). Increasing the lifetime and a more intensive use of material stocks, as envisaged via increasing value and utility of products, are important measures towards a CE and need to be developed in this direction. Additionally, improved recycling technology and changes in product design to enhance recyclability are important challenges (Ciacci et al. 2015; Reck and Graedel 2012; Gaustad et al. 2010). Clearly, also a reduction of fossil fuel consumption is urgently required to mitigate climate change, which would also increase socioeconomic circularity (Ghisellini et al. 2016). For renewable biomass resources, CE strategies should focus on less wasteful, more efficient, and cascadic uses as well as a production system that fosters and sustains ecological cycles, rather than simply increasing biomass inputs to substitute other materials (Smith et al. 2014; Haberl and Geissler 2000).

### Further Advancing Data and Indicators for Circular Economy Assessments

We introduced an economy‐wide and mass‐based framework to monitor progress towards a CE and shown that data from ew‐MFA and waste statistics can be used to calculate a set of headline indicators. Our research also revealed a number of limitations and weaknesses, which need to be addressed to better exploit the potential of this approach and to provide better and more reliable assessments of a CE in the future.

#### Improving Statistical Reporting

Consistently integrating data from different statistical sources reveals the need to improve statistical reporting and to explicitly address uncertainties in reported quantities. Similar to other statistical data sources, it would be beneficial to develop an official concordance between ew‐MFA and waste statistics. In particular, the quality of waste statistics calls for improvements, since it is unclear how complete the coverage of waste flows in the different countries is and reported data seem to be of very different quality. Discrepancies between the amount of recovered and actually recycled materials replacing primary material are not well understood and reported and lead to an overestimation of socioeconomic cycling rates. Schiller and colleagues (2017) conclude that for Germany, only 48% of outflows of concrete and bricks are suitable for high‐quality recycling, reducing the recycling share in fresh concrete to a maximum of 32%. A Swiss study of polyethylene terephthalate (PET) bottles, tinplate, aluminum, paper and cardboard, and glass reveals that actual recycling rates are substantially lower than reported in official statistics (Haupt et al. 2017), and the authors expect similar differences in other countries. Thus, the EU28’s continuous efforts towards a CE calls for improvements and harmonization in statistical reporting.

Improving knowledge about material stocks would enable filling gaps in waste statistics and cross‐validating mass balances, explicitly considering so‐called hibernating stocks, and gaining insights about the future dynamics of EoL waste potentials. Currently, waste statistics (incompletely) report those end‐of‐life materials which are collected and enter waste treatment facilities. On‐site waste flows often downcycled are not covered, as are so‐called hibernating stocks (Hashimoto et al. 2009), which results in an overestimation of NAS and an underestimation of outflows from stocks, and adds considerable uncertainty about indicators for socioeconomic loop closing. Lack of knowledge about the aging dynamics of in‐use stocks means that future EoL flows cannot be prospectively modeled, nor can spatial patterns of waste flows be derived. Such information would be useful to inform waste management planning and circularity potentials. Closing the knowledge gap about in‐use stocks requires records of in‐use vintages of material stocks and their service lifetimes, which can be achieved via different modeling approaches, depending on data availability (Miatto et al. 2017; Augiseau and Barles 2017; Fishman et al. 2014; Pauliuk et al. 2012).

#### Broadening the Scope of Circular Economy Monitoring

The proposed framework provides a set of mass‐based indicators for monitoring the progress towards a more circular economy in terms of material loop closing. The strength of this approach is the system‐wide perspective, which provides a comprehensive picture of material loop closing in a CE across all materials used by society. The proposed indicators allow us to detect problem shifting and can be used to determine target values to downscale the industrial metabolism (Bringezu 2015), which, in addition to targeted measures for specifically problematic flows, must be the ultimate aim of a circular economy. The aggregate indicators, however, obscure flows of individual materials or substances, which may be of high interest for CE policies due to criticality or specific environmental pressures but have only a low share in terms of mass. Plastics, for example, are subsumed under fossil energy carriers or critical metals (European Commission 2010) under metal ores and metals. Tracing changes in these flows requires additional data, which can be obtained from more detailed material flow analysis (Nuss and Blengini 2018; Brooks et al. 2018) and should be integrated consistently into the proposed framework. The mass‐based indicators deliver a robust data basis that is extendable with more in‐depth assessments of individual materials, but also with social, economic, and environmental indicators. For example, Van der Voet and colleagues (2009) proposed an environmentally weighted material consumption indicator (EMC) to measure the total environmental impact of material flows, and Pauliuk (2018) proposed a dashboard of CE core indicators for organizations that partly builds upon MFA methods. Additionally, a more explicit measurement of energy flows (Cleveland et al. 2000) or differences in energy quality (Ayres and Warr 2009) can be modeled based on this framework. Also, a better understanding of the GHG implications of ecological cycling is required.

#### Criteria for Ecological Loop Closing

Currently, it is assumed that all used biomass contributes to ecological loop closing; this is the case only if biomass production and discharge maintains the regenerative capacities of eco‐systems. A proper assessment of ecological loop closing is, however, not possible due to a lack of data and appropriate indicators. Criteria to be considered refer to soil degradation, overexploitation of water resources, efficient management of plant nutrients and livestock manure (Zanten et al. 2018; Dawson and Hilton 2011), the regional scale of ecological loop closing (Therond et al. 2017), and net carbon losses related to cultivation (Erb et al. 2017; Fargione et al. 2008). This issue is of particular importance because climate change mitigation and bioeconomy strategies foster the use of biomass.

#### Global Integration

Our assessment focuses on material flows and circularity within the EU28; however, as trade with waste and secondary resources is rapidly increasing, it is important to also capture the global cross‐country dimension of circularity in national assessments (Beukering 2013; Berglund and Söderholm 2003; Frazzoli et al. 2010). While consumption‐based indicators such as the RMC capture the global dimension of domestic final consumption (Wiedmann et al. 2015), exports and imports of waste and secondary materials must be better documented and integrated in the assessment framework and derived indicators.

### Conclusions and the Way Forward

The concept of a CE is a powerful bridging concept to foster the fundamental links among resource use, waste, and emissions, and to contribute to integrating environmental (output‐related) and economic (input‐related) policies. Environmental pressures resulting from the scale and structure of the industrial metabolism require concerted action on both ends. Improved collaboration between these currently isolated policy domains could realize co‐benefits among environment, employment, and security of supply. Beyond the policy arena, well‐attuned concerted actions of policy makers with industries ranging from production to waste management is a further necessary strand for pushing the CE. At the same time, all these initiatives require monitoring frameworks, which provide indicators to assess links between a CE and sustainability goals on all scales. The combination of a systematic and mass‐balanced approach with regularly published statistical data calls for improvements of data quality, standardizing waste statistics, and consolidating them with ew‐MFA data. Such improvements allow better understanding of not only the level of circularity, but the quality of the circularity as well, which would, in turn, allow for a better understanding of the true contribution to sustainability goals.

## Supplementary Information

**Supporting Information S1**: This supporting information provides details of all assumptions, primary and secondary data sources, and various calculation factors referred to in the article.

**Supplementary Data S2**: This supporting information provides details of all data referred to in the article, along with a complete system description. Color coding in this SI file is as follows: green = data as used in figures; yellow = final circular economy system description; blue = calculation steps; red = raw data files as downloaded.

## Footnotes

1 Gigatons and tons refer to *metric tons* throughout the manuscript and supporting information files.
