# Supplementary material for: Measuring Progress towards a Circular Economy: A Monitoring Framework for Economy‐wide Material Loop Closing in the EU28
Source: J Ind Ecol. 2018 Sep 25;23(1):62–76. doi: 10.1111/jiec.12809 (PMC6472471; doi:10.1111/jiec.12809)
Supplement: Supplementary file 1 — Supporting Information S1: This supporting information provides details of all assumptions, primary and secondary data sources, and various calculation factors referred to in the article. [file 44498_2019_Article_2301005_MOESM1_ESM.pdf]

SUPPORTING INFORMATION FOR:

Mayer, A., W. Haas, D. Wiedenhofer, F. Krausmann, P. Nuss, and G.A. Blengini. 2018. Measuring progress towards a Circular Economy: A monitoring framework for economy-wide material loop closing in the EU28. *Journal of Industrial Ecology.*

Summary

This supporting information S1 provides details of all assumptions, primary and secondary data sources and various calculation factors referred to in the article.

Table S1: Material flows and model variables for the calculation of indicators of economy wide circularity. Model variables are listed according their appearance in figure 1 from left to right. 2

Figure S1: Material flows through the EU28 economy in 2014 with more detailed input flows in Gt/yr. In this Sankey diagram, the width of the arrows is proportional to the size of material flows (dark blue). DMI = Direct material input, DMC = Domestic material consumption. 4

Table S2: Data sources and assumptions used to quantify the main material flows by material groups 5

Table S3: Detailed results of the one-at-a-time sensitivity analysis for three CE indicators (PM, ISCr, OSCr) and three material flows (NAS, dem&disc, DPOe). 6

Table S4: Waste flow codes (W-Code) for NACE waste categories as reported by Eurostat and allocation to main ew-MFA categories and material or energetic use. Source: env_wastrt accounts (Eurostat, 2017a) 7

Table S5: Comparison calculated emissions from fossil fuel combustion with data reported in emission statistics (Eurostat, 2017d) 8

Table S6: Empirical results of main material flows and CE indicators for the EU28 in 2014 in tons and %. 10

Table S7: Comparison of main and auxiliary CE indicators from Haas et al. (2015) with this study (2014). 11

References 12

Table S1: Material flows and model variables for the calculation of indicators of economy wide circularity. Model variables are listed according their appearance in figure 1 from left to right.

Model variable	Sources, calculation	Description
Import	Material flow database (Eurostat, 2017a)	Extra EU imports
Export	Material flow database (Eurostat, 2017a)	Extra EU exports
Domestic extraction (DE)	[Material flow database (Eurostat, 2017a)	Materials extracted from within the EU28 domestic environment
Domestic material consumption (DMC)	DMC = DE + import - export	Apparent consumption of materials
Re- and downcycling, i.e. secondary materials (SM)	Waste statistics (Eurostat, 2017b)	Re- and downcycling comprises flows reported as recycling or backfilling in waste statistics, where. recycling is defined as 'any recovery operation by which waste materials are reprocessed into products, materials or substances whether for the original or other purposes' and backfilling as a recovery operation where waste is used in excavated areas (such as underground mines, gravel pits) and where the waste is substituting other non-waste materials which would have had to be used for the purpose. We refer to re- and downcycling also as secondary materials.<br>Flows that are not consistent with ew-MFA system boundaries (i.e. dredging spoils, soils) were excluded.
Processed materials (PM)	DMC + SM	All primary and secondary materials used in the socio-economic system.
Energetic use (eUse)	Calculated from material flow database and information from (Faostat, 2017; IEA, 2017)	Fraction of PM that is used to provide energy. eUse comprises not only technical energy but also feed for livestock and food for humans.
Material use (mUse)	Calculated (see SI)	Fraction of PM that is used for material purposes. Comprises all metals and non metallic minerals, fractions of biomass and fossil energy carriers.
Throughput materials	MFA database<br>Waste statistics	Short-lived products (life span <1 yr) and processing and manufacturing waste (recorded in waste statistics); wastage and deliberative

	Estimates	dissipative uses (not recorded in waste statistics).
Extractive waste	Calculated from DE of ores (Eurostat, 2017a)	Waste rock from domestic mining
(Gross) additions to stocks (GAS)	Calculated	Materials used to build up in-use stocks of materials (life span >1 yr).
Demolition & discard	Calculated based on waste statistics	Solid waste from discarded in-use stocks (comprises construction and demolition waste but also all other discarded long living products
Solid waste from energetic use	Waste statistics (Eurostat, 2017b) and calculated	Solid waste from combustion of fuels and excrements of humans and livestock at the water content of biomass intake (i.e. excluding water uptake by humans and livestock)
EoL waste	Waste statistics (Eurostat, 2017b) and calculated	Total end of life waste comprises all solid waste from eUse and mUse including throughput materials and extractive waste.
Interim outputs (IntOut)	Eol waste + emissions	All wastes and emissions after the use phase.
DPO emissions (DPOe)	Calculated and cross checked with Eurostat emission statistics	All gaseous outputs including vapor from combustion and human and animal respiration; oxygen input from air is excluded.
DPO waste (DPOw)	DPOw = EoL waste - SM	All EoL waste excluding materials recovered for re- and downcycling. All liquid and solid outputs including moisture content as included in extracted material but excluding extra added water (e.g during industrial processes or drinking water).
DPO	DPOe + DPOw	Total domestic processed output to the environment (waste and emissions).

Figure S1: Material flows through the EU28 economy in 2014 with more detailed input flows in Gt/yr. In this Sankey diagram, the width of the arrows is proportional to the size of material flows (dark blue). DMI = Direct material input, DMC = Domestic material consumption.

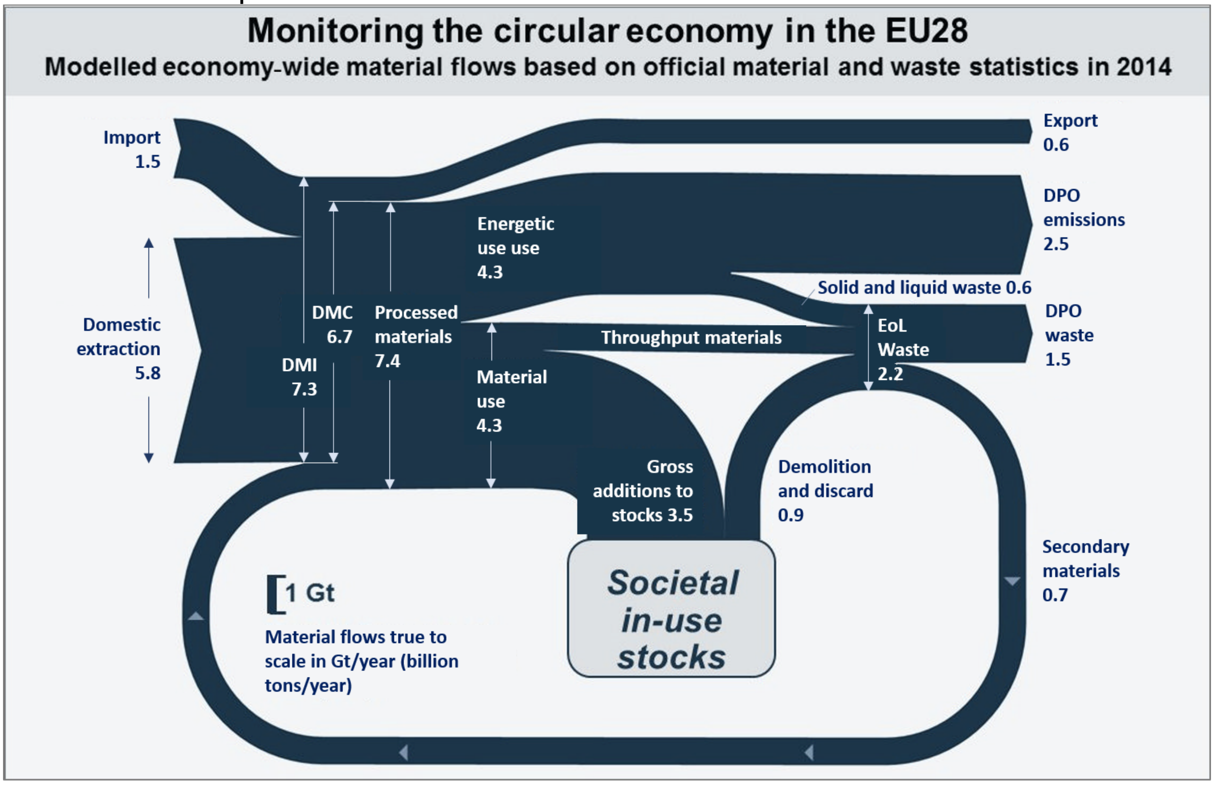

Table S2: Data sources and assumptions used to quantify the main material flows by material groups

Main material group	Allocation to material or energy use	Allocation of material use to additions to stock or throughput materials
**Biomass**	Crops based on FAO food balance sheets; “other uses” in FAO food balance sheets are assigned to material use minus renewable energies (Eurostat, 2017c); data cross checked with Krausmann et al. (2008);	No additions to stocks
	Crop residues equally to material and energy use	Crop residues (straw) assumed 10% stock add based on Haas et al. (2015)
	Wood: Timber allocated to material use and wood fuel to energetic use based on Haas et al. (2015)	Timber (Industrial roundwood) as stock add except for paper production assumed as 10% of roundwood based on Haas et al. (2015) and Krausmann et al. (2008, 2017)
**Fossil energy carriers**	Fossil energy assigned to energetic use with the following exceptions: plastics based on Plastics Europe (2015); tyres based on Shulman (2011); lubricants based on Monier, Veronique and Labouze (2001). Fertilizer based on Wood and Cowie (2004)	Stock add to throughput split for plastics based on Plastics Europe (2015); tyres on Shulman (2011); lubricants on Monier, Veronique and Labouze (2001).
**Metals (i.e. metal content)**	100% mUse, except Uranium and Thorium	Weighted averages based on data for Iron: Wang et al. (2007); Aluminum: Cullen and Allwood (2013); Other metals: (Allwood et al., 2010)
**Extractive waste (tailings)**	Directly ascribed to interim outputs	Directly ascribed to interim outputs
**Industrial minerals**	100% mUse	Salt and fertilizers allocated to throughput materials and slate mainly (95%) to additions to
**Construction minerals**	100% mUse	Ascribed to additions to stocks except for a 5% flow assumed to be throughput materials (wastes and losses during construction) see (Krausmann et al., 2017)

Table S3: Detailed results of the one-at-a-time sensitivity analysis for three CE indicators (PM, ISCr, OSCr) and three material flows (NAS, dem&disc, DPOe).

	size or rate Indicator (% or Gt)									change of indicator (% to standard run)							
	PM	ISCr	OSCr	DPOe	IntOut	DPO	NAS	demolition and discard		PM	ISCr	OSCr	DPOe	IntOut	DPO	NAS	demolition and discard
**Baseline**	7,400,945	10%	15%	2,625,800	4,775,145	4,067,173	2,625,800	928,344		0.0%	0.0%	0.0%	0.0%	0.0%	0.0%	0.0%	0.0%
**ew-MFA +20%**	8,739,540	8%	13%	3,314,257	5,425,282	4,717,310	3,314,257	861,782		18.1%	-15.3%	-12.0%	26.2%	13.6%	16.0%	26.2%	-7.2%
**ew-MFA -20%**	6,062,350	12%	17%	1,937,342	4,125,008	3,417,036	1,937,342	994,905		-18.1%	22.1%	15.8%	-26.2%	-13.6%	-16.0%	-26.2%	7.2%
**env_wastrt +20%**	7,542,539	11%	17%	2,419,141	5,123,399	4,273,832	2,419,141	1,218,568		1.9%	17.7%	11.8%	-7.9%	7.3%	5.1%	-7.9%	31.3%
**env_wastrt -20%**	7,259,351	8%	13%	2,832,458	4,426,892	3,860,515	2,832,458	638,120		-1.9%	-18.4%	-13.7%	7.9%	-7.3%	-5.1%	7.9%	-31.3%
**SM +20%**	7,542,539	9%	15%	2,725,394	4,817,145	3,967,579	2,725,394	912,563		1.9%	-1.9%	-0.9%	3.8%	0.9%	-2.4%	3.8%	-1.7%
**SM -20%**	7,259,351	10%	15%	2,526,205	4,733,145	4,166,768	2,526,205	944,125		-1.9%	2.0%	0.9%	-3.8%	-0.9%	2.4%	-3.8%	1.7%
**mUse +20% / eUse -20%**	7,400,945	10%	15%	2,694,944	4,706,001	3,998,029	2,694,944	903,598		0.0%	0.0%	1.5%	2.6%	-1.4%	-1.7%	2.6%	-2.7%
**mUse -20% / eUse +20%**	7,400,945	10%	15%	2,556,655	4,844,290	4,136,318	2,556,655	953,089		0.0%	0.0%	-1.4%	-2.6%	1.4%	1.7%	-2.6%	2.7%
**Biomass +20%**	7,400,945	10%	15%	2,601,837	4,799,108	4,091,136	2,601,837	929,148		0.0%	0.0%	-0.5%	-0.9%	0.0%	0.0%	-0.9%	0.1%
**Biomass -20%**	7,400,945	10%	15%	2,649,763	4,751,182	4,043,210	2,649,763	927,540		0.0%	0.0%	0.5%	0.9%	0.0%	0.0%	0.9%	-0.1%
**Metals and ores +20%**	7,400,945	10%	15%	2,634,941	4,766,004	4,058,032	2,634,941	925,616		0.0%	0.0%	0.2%	0.3%	0.0%	0.0%	0.3%	-0.3%
**Metals and ores -20%**	7,400,945	10%	15%	2,616,658	4,784,287	4,076,315	2,616,658	931,071		0.0%	0.0%	-0.2%	-0.3%	0.0%	0.0%	-0.3%	0.3%
**Industrial minerals +20%**	7,400,945	10%	15%	2,628,476	4,772,469	4,064,497	2,628,476	925,110		0.0%	0.0%	0.1%	0.1%	0.0%	0.0%	0.1%	-0.3%
**Industrial minerals -20%**	7,400,945	10%	15%	2,623,123	4,777,822	4,069,850	2,623,123	931,577		0.0%	0.0%	-0.1%	-0.1%	0.0%	0.0%	-0.1%	0.3%
**Fossil energy carriers +20%**	7,400,945	10%	15%	2,615,670	4,785,275	4,077,303	2,615,670	929,919		0.0%	0.0%	-0.2%	-0.4%	0.0%	0.0%	-0.4%	0.2%
**Fossil energy carriers -20%**	7,400,945	10%	15%	2,635,929	4,765,016	4,057,044	2,635,929	926,769		0.0%	0.0%	0.2%	0.4%	0.0%	0.0%	0.4%	-0.2%
**Construction minerals +20%**	7,400,945	10%	15%	2,654,048	4,746,897	4,038,925	2,654,048	933,859		0.0%	0.0%	0.6%	1.1%	0.0%	0.0%	1.1%	0.6%
**Construction minerals -20%**	7,400,945	10%	15%	2,597,551	4,803,394	4,095,422	2,597,551	922,829		0.0%	0.0%	-0.6%	-1.1%	0.0%	0.0%	-1.1%	-0.6%

Table S4: Waste flow codes (W-Code) for NACE waste categories as reported by Eurostat and allocation to main ew-MFA categories and material or energetic use. Source: env_wastrt accounts (Eurostat, 2017a)

The conceptual gap between waste statistics and ew-MFA is based on different reporting classifications, and we have allocated waste flows to ew-MFA categories based on the main material components of waste flows. This was only feasible at the level of the main material groups distinguished in ew-MFA, i.e. biomass, fossil energy carriers, industrial minerals, construction minerals, metal ores. Table S3 shows detailed allocations of each waste flow to the aforementioned ew-MFA main material categories, and whether waste flows stem from eUse or mUse. Expert informed assumptions were necessary to judge whether waste flows reported in statistics result from energetic or material use. Most waste flows could unambiguously be allocated to wastes from material use. Among the waste flows originating from energetic use were animal and vegetal wastes (W09) and combustion wastes (W124). A few flows recorded in waste statistics (e.g.) were excluded, since they are follow a different system boundary and thus are not recorded as extraction in ew-MFA statistics. The quantitatively most important flows that were excluded were soils (W126) and dredging spoils (W127). For a detailed allocation of waste flows to ew-MFA categories, see table below.

Legend: WASTE/WST_OPER = Waste category, Exclude from calculations = Exclusion of individual categories to eliminate double-counting, aggregated categories, and alignment with ew-MFA system boundaries, Biomass, Metals, Industrial minerals, Fossils, Construction = Share of individual waste flow categories in ew-MFA main material category, Total = Sum of shares, Material use = Share of waste stemming from material use, Energetic use = share of waste stemming from energetic use

WASTE/WST_OPER	Exclude from calculations	Biomass	Metals	Industrial minerals	Fossils	Construc tion	Total	material use	energetic use
TOTAL - Total Waste	Aggregate	-	-	-	-	-	-	-	-
**W01-05 - Chemical and medical wastes (subtotal)**	Aggregate	-	-	-	-	-	-	-	-
W011 - Spent solvents	-	0,30	-	0,40	0,30	-	1	1	-
W012 - Acid, alkaline or saline wastes	-	1,00	-	-	-	-	1	1	-
W013 - Used oils	-	-	-	-	1,00	-	1	1	-
W02A - Chemical wastes	-	0,40	-	0,30	0,30	-	1	1	-
W032 - Industrial effluent sludges	-	0,50	-	0,50	-	-	1	1	-
W033 - Sludges and liquid wastes from waste treatment	Double-counting	-	-	-	-	-	-	-	-
W05 - Health care and biological wastes	-	1,00	-	-	-	-	1	1	-
**W06_07A - Recyclable wastes (subtotal, W06+W07 except W077)**	Aggregate	-	-	-	-	-	-	-	-
W061 - Metal wastes, ferrous	included in W06 - Metal	-	-	-	-	-	-	-	-
W062 - Metal wastes, non-ferrous	included in W06 - Metal	-	-	-	-	-	-	-	-
W063 - Metal wastes, mixed ferrous and non-ferrous	included in W06 - Metal	-	-	-	-	-	-	-	-
W071 - Glass wastes	-	-	-	1,00	-	-	1	1	-
W072 - Paper and cardboard wastes	-	1,00	-	-	-	-	1	1	-
W073 - Rubber wastes	-	0,06	0,20	-	0,75	-	1	1	-
W074 - Plastic wastes	-	-	-	-	1,00	-	1	1	-
W075 - Wood wastes	-	1,00	-	-	-	-	1	1	-
W076 - Textile wastes	-	1,00	-	-	-	-	1	1	-
**W077_08 - Equipment (subtotal, W077+W08A+W081+W0841)**	Aggregate	-	-	-	-	-	-	-	-
W077 - Waste containing PCB	-	-	0,50	0,50	-	-	1	1	-
W08A - Discarded equipment (except discarded vehicles and batteries and accumulators waste) (W08 except W081, W0841)	-	-	1,00	-	-	-	1	1	-
W081 - Discarded vehicles	-	-	1,00	-	-	-	1	1	-
W0841 - Batteries and accumulators wastes	-	-	1,00	-	-	-	1	1	-
**W09 - Animal and vegetal wastes (subtotal, W091+W092+W093)**	Aggregate	-	-	-	-	-	-	-	-
W091 - Animal and mixed food waste	included inW091_092 -	-	-	-	-	-	-	-	-
W092 - Vegetal wastes	included inW091_092 -	-	-	-	-	-	-	-	-
W093 - Animal faeces, urine and manure	Double-counting	-	-	-	-	-	-	-	-
**W10 - Mixed ordinary wastes (subtotal, W101+W102+W103)**	Aggregate	-	-	-	-	-	-	-	-
W101 - Household and similar wastes	-	0,68	0,09	0,12	0,04	0,07	1	1	-
W102 - Mixed and undifferentiated materials	-	0,68	0,09	0,12	0,04	0,07	1	1	-
W103 - Sorting residues	-	0,68	0,09	0,12	0,04	0,07	1	1	-
W11 - Common sludges	-	1,00	-	-	-	-	1	-	1
**W12-13 - Mineral and solidified wastes (subtotal)**	Aggregate	-	-	-	-	-	-	-	-
W121 - Mineral waste from construction and demolition	-	0,11	0,05	0,05	0,00	0,79	1	1	-
W12B - Other mineral wastes (W122+W123+W125)	-	0,11	0,05	0,05	0,00	0,79	1	1	-
W124 - Combustion wastes	-	-	-	-	1,00	-	1	-	1
W126 - Soils	Exclude	-	-	-	-	-	-	-	-
W127 - Dredging spoils	Exclude	-	-	-	-	-	-	-	-
W128_13 - Mineral wastes from waste treatment and stabilised wastes	Double-counting	-	-	-	-	-	-	-	-
**W06 - Metallic wastes (W061+W062+W063)**	-	-	1,00	-	-	-	1	1	-
W091_092 - Animal and mixed food waste; vegetal wastes (W091+W092)	-	1,00	-	-	-	-	1	-	1
W11_127 - Common sludges and dredging spoils (W11+W127, valid up to 2008)	-	0,50	-	-	-	0,50	1	-	-
W12_X_127NH - Mineral waste (except non-hazardous dredging spoils, valid up to 2008)	-	0,11	0,05	0,05	0,00	0,79	1	1	-
RCV_OTH - Other recovered wastes (valid up to 2008)	-	0,20	0,20	0,20	0,20	0,20	1	-	-
DSP_OTH - Other disposed wastes (valid up to 2008)	-	-	0,33	0,33	-	0,33	1	-	-
INC_OTH - Other incinerated wastes (valid up to 2008)	-	0,80	-	-	0,20	-	1	-	-
TOT_X_MIN - Waste excluding major mineral wastes	Aggregate	-	-	-	-	-	-	-	-

Table S5: Comparison calculated emissions from fossil fuel combustion with data reported in emission statistics (Eurostat, 2017d)

**Emission statistics**	Value (Mt)
CO_2	3,724
C	1,015
CH_4	18
N_2O	1
SO_2	4
S	2
Sum excluding oxygen from air	*1,036*
**This study**	
Fossil fuels (wet)	1,445
Average vapor including vapor from combustion and excess H_2 (%)	24%
Fossil fuels (DM)	*1,099*
**Comparison**	
Difference ew-MFA – emission accounts (t)	63
Difference ew-MFA – emission accounts (%)	5.7%

Our calculation includes all emissions to air from biomass including all gaseous outputs from humans and livestock which are only partly included in emission statistics. Therefore. we focused in our comparison on emissions to air from fossil fuels.

We performed the following steps:

- We extracted the following emission to air from Eurostat (2017c) for 2014
    - Year 2014. all NACE branches plus households in tonnes for CO_2. CH_4. N_2O and SO_2.
- We converted the emissions into the chemical elements contained in the fuel at the point of extraction. Thus. CO_2 was converted to C and SO_2 to S. CH_4 and N_2O are emissions stemming from elements already included in fossil fuels.
- We extracted DMC fossil fuel data from our calculation files (originally from ew-MFA statistics) and deducted the vapor generated during combustion (moisture content. H_2O built from elements contained in fuel and excess H_2). We assume standard moisture content for brown coal. hard coal. oil shale and tar sands. peat. crude oil. condensate and natural gas liquids. natural gas and fuel for land. water and air transport.
- Finally. we compared fossil fuel emissions from dry matter (DM) derived from our calculation with emissions to air from emission accounts excluding oxygen from air. Table SY presents the results.
- The difference is that our calculation is about 5.7% higher than the emission accounts which seems to be reasonable.

Table S6: Empirical results of main material flows and CE indicators for the EU28 in 2014 in tons and %.

	**Non-metallic minerals**	**Fossil energy carriers**	**Biomass**	**Metal ores and metals**	**Extractive waste**	**Totals**
**DE**	3,071,343	723,001	1,801,860	20,196	167,998	**5,784,397**
**DMC**	3,050,169	1,555,927	1,817,205	101,674	167,998	**6,692,973**
**Import**	77,338	1,061,349	184,856	214,286		**1,537,828**
**Export**	98,512	228,423	169,511	132,808		**629,253**
**PM**	3,371,382	1,620,876	2,016,203	224,487	167,998	**7,400,945**
**eUse**		1,567,206	1,570,497	79		**3,137,781**
**mUse**	3,371,382	53,670	445,706	224,408		**4,263,164**
**GAS**	3,128,164	25,595	221,993	178,392		**3,554,143**
**Demolition and discard**	706,697	3,261	88,415	129,970		**928,344**
**NAS**	2,421,467	22,333	133,578	48,422		**2,625,800**
**Throughput materials**	243,218	28,075	223,713	46,016	167,998	**709,020**
**recycling**	272,644	56,558	191,338	119,776		**640,316**
**backfilling**	48,569	8,391	7,660	3,037		**67,656**
**SM**	321,219	64,953	199,025	122,815		**708,012**
**Waste mUse**	949,915	31,337	312,128	175,986	167,998	**1,637,364**
**Waste eUse**		121,310	454,739			**576,049**
**EoL waste**	949,915	152,647	766,868	175,986	167,998	**2,213,413**
**DPOw**	628,703	87,697	567,870	53,173	167,998	**1,505,441**
**DPOe**		1,445,896	1,115,757	79		**2,561,732**
**Interim Outputs**	949,915	1,598,543	1,882,625	176,065	167,998	**4,775,145**
**DPO total**	628,703	1,533,593	1,683,627	53,252	167,998	**4,067,173**
**ISCr[1]**	9.5%	4.0%	9.9%	31.3%[3]/54.7%[4]	n.a.	**9.6%**
**OSCr[2]**	33.8%	4.1%	10.6%	35.7%[3]/69.8%[4]	n.a.	**14.8%**
**IECrp**	n.a.	n.a.	n.a.	n.a.	n.a.	**24.6%**
**OECrp**	n.a.	n.a.	n.a.	n.a.	n.a.	**35.3%**
**INCr**	n.a.	n.a.	n.a.	n.a.	n.a.	**21.2%**
**ONCr**	n.a.	n.a.	n.a.	n.a.	n.a.	**32.8%**
**Material recovery[5]**	33.8%	42.5%	25.9%	35.7%[3]/69.8%[4]	n.a.	**32.0%**

Legend:
[1] Input socio-economic cycling rates per material category are calculated as (recycling+backfilling per main material category)/PM per main material category
[2] Output socio-economic cycling rates per material category are calculated as (recycling+backfilling per main material category)/Interim outputs per main material category
[3] Rates including extractive waste
[4] Rates excluding extractive waste
[5] Material recovery = (recycling+backfilling)/EoL waste

Table S7: Comparison of main and auxiliary CE indicators from Haas et al. (2015) with this study (2014)

	Haas et al. (2015) for EU27 in 2005	This study for EU28 in 2014
DMC (Gt)	6.7	6.7
DPO (Gt)	5.0	4.1
PM (Gt)	7.7	7.4
ISCr (%)	13%	10%
IECrp (%)	28%	25%
Demolition and discard (Gt)	1.7	0.9
INCr	26%	21%

The lower demolition and discard waste flows in our results compared to Haas et al. cause a lower DPO and a lower ISCr. Lower results are mainly due to statistical life times in Haas et al. (2015) that turn stocks out of service in the calculation into waste flows while our results only used flows reported in waste statistics. Thus, hibernating stocks become waste in Haas et al. and remain as unused stock in the stocks in our calculation.

References

Allwood, J.M., Cullen, J.M., Milford, R.L., 2010. Options for Achieving a 50% Cut in Industrial Carbon Emissions by 2050. Environ. Sci. Technol. 44, 1888–1894. https://doi.org/10.1021/es902909k

Cullen, J.M., Allwood, J.M., 2013. Mapping the global flow of aluminum: from liquid aluminum to end-use goods. Environ. Sci. Technol. 47, 3057–3064. https://doi.org/10.1021/es304256s

Eurostat, 2017a. Material flow accounts (env_ac_mfa) [WWW Document]. URL http://appsso.eurostat.ec.europa.eu/nui/show.do?dataset=env_ac_mfa&lang=en

Eurostat, 2017b. Waste generation and treatment (env_wastrt) [WWW Document]. URL http://appsso.eurostat.ec.europa.eu/nui/show.do?dataset=env_wastrt&lang=en

Eurostat, 2017c. Supply, transformation and consumption of renewable energies [nrg_1007a] [WWW Document].

Eurostat, 2017d. Air emissions accounts totals bridging to emission inventory totals [env_ac_aibrid_r2] [WWW Document].

Faostat, 2017. FAOSTAT database [WWW Document]. URL http://www.fao.org/faostat/en/ (accessed 1.13.16).

Haas, W., Krausmann, F., Wiedenhofer, D., Heinz, M., 2015. How Circular is the Global Economy?: An Assessment of Material Flows, Waste Production, and Recycling in the European Union and the World in 2005. Journal of Industrial Ecology 765–777. https://doi.org/10.1111/jiec.12244

IEA, 2017. A world in transformation: World Energy Outlook 2017. International Energy Association, Paris.

Krausmann, F., Erb, K.H., Gingrich, S., Lauk, C., Haberl, H., 2008. Global patterns of socioeconomic biomass flows in the year 2000: A comprehensive assessment of supply, consumption and constraints. Ecological Economics 65, 471–487.

Krausmann, F., Wiedenhofer, D., Lauk, C., Haas, W., Tanikawa, H., Fishman, T., Miatto, A., Schandl, H., Haberl, H., 2017. Global socioeconomic material stocks rise 23-fold over the 20th century and require half of annual resource use. PNAS 201613773. https://doi.org/10.1073/pnas.1613773114

Monier, Veronique, Labouze, E., 2001. 'Critical Review Of Existing Studies And Life Cycle Analysis On The Regeneration And Incineration Of Waste Oils'. Taylor Nelson Sofres S. A., Montrouge cedex.

Plastics Europe, 2015. Plastic - The facts (2015). Plastics Europe, Bruxelles.

Shulman, V., 2011. Tyre Recycling, in: WASTE. A Handbook of Waste Management and Recycling. Elsevier, Amsterdam, pp. 297–320.

Wang, T., Müller, D.B., Graedel, T.E., 2007. Forging the anthropogenic iron cycle. Environmental science & technology 41, 5120–5129.

Wood, S., Cowie, A., 2004. A review of greenhouse gas emission factors for fertiliser production. Report for International Energy Agency (IEA) Bioenergy Task 38. Research and Development Division, State Forests of New South Wales.
